# Persistence of Immunity in Adults after 1, 5 and 10 Years with Recombinant Hepatitis B Vaccine in Beijing in 2010–2020

**DOI:** 10.3390/vaccines10020181

**Published:** 2022-01-25

**Authors:** Sijia Shen, Shen Ge, Zheng Zhang, Jianxin Ma, Yang Jiao, Qian Li, Yan Liang, Shuming Li

**Affiliations:** 1 School of Public Health, Shanxi Medical University, Taiyuan 030001, China; cyqwjwcdcywbgs@bjchy.gov.cn; 2 Chaoyang District Center for Disease Prevention and Control of Beijing, Beijing 100020, China; cyqwjwcdcgs@bjchy.gov.cn (S.G.); cyqwjwcdczz@bjchy.gov.cn (Z.Z.); cyqwjwcdcmjx@bjchy.gov.cn (J.M.); cyqwjwcdcjy@bjchy.gov.cn (Y.J.); cyqwjwcdclq@bjchy.gov.cn (Q.L.); cyqwjwcdcly@bjchy.gov.cn (Y.L.)

**Keywords:** hepatitis B vaccine, adult, immune persistence

## Abstract

The persistence of immunity after hepatitis B vaccination is still under investigation in adults. In Chaoyang District, Beijing, people who were aged ≥ 18 years and completely immunized with HBV vaccine according to the standard procedure (0–1–6 months) were enrolled. Three groups were set for 1 (Y1), 5 (Y5) and 10 (Y10) years after the hepatitis B vaccination. The following data was collected and analyzed: antibody against hepatitis B virus surface antigen(anti-HBs) positive rates and geometric mean concentration (GMC) between the different compared groups through questionnaires and laboratory detection, including hepatitis B virus surface antigen (HBsAg), anti-HBs and antibody against hepatitis B virus core antigen(anti-HBc). All 600 subjects completed the questionnaires and serological tests. Among all subjects, the positive rates of HBsAg, anti-HBs and anti-HBc were 0, 70.5% (423/600) and 2.5% (15/600), respectively. The anti-HBs positive rates in Y1, Y5 and Y10 groups were 86.5% (173/200), 71.0% (142/200) and 54.0% (108/200) (χ^2^ = 50.8, *p* < 0.001) and showed a linear decreasing trend year by year (trend χ^2^ = 50.7, *p* < 0.001). The GMC in Y1, Y5 and Y10 groups were 296.6 mIU/mL, 51.6 mIU/mL and 25.5 mIU/mL (H = 64.8, *p* < 0.001), respectively. The anti-HBs positive rates and GMC decreased rapidly after the vaccination of adults against hepatitis B. Screening after 5–10 years and booster vaccination for the unprotected population is recommended.

## 1. Introduction

Hepatitis B is caused by infection with the Hepatitis B virus (HBV) and can lead to a wide spectrum of liver disease ranging from acute hepatitis to chronic hepatitis [1]. The symptoms and outcome during acute HBV infection depend on age at infection. Infants and children are mostly asymptomatic, while roughly 70% of adults have subclinical or anicteric hepatitis and 30% have icteric hepatitis [2]. Chronic hepatitis B has a variable and dynamic course, including immune tolerance phase, immune clearance phase, inactive carrier phase and reactivating phase and can lead to cirrhosis and hepatocellular carcinoma (HCC) [1,3]. Globally, in 2015, there were more than 2 billion people infected with HBV, of which 257 million were living with chronic HBV infection and 1.19 million people died from HCC and cirrhosis [4]. The annual direct and indirect medical costs of hepatitis B in China amounted to RMB 260 billion, and the treatment of 20 million chronic hepatitis B cases costs around $9.5 billion per year [5].

The hepatitis B vaccine has been proved very effective at preventing infection with HBV [6,7]. Currently, the recombinant hepatitis B vaccine is widely used in China for immunization; it can be classified as Hansenula (HepB-HP), Saccharomyces cerevisiae (HepB-SC) and Chinese hamster ovary cells (HepB-CHO), and the dosage forms can be classified as 5 μg/dose, 10 μg/dose, 20 μg/dose and 60 μg/dose. In China, hepatitis B vaccination in infants was implemented by the Chinese government as a basic state policy in 2002 and free for newborns in 2005 [8]. This way, the prevalence of HBV in children aged 0–4 years (0.3%) decreased by 96.7% in 2014 compared to 1992 (9.7%). However, in adults, it has not decreased significantly [9]. Currently, new cases of acute hepatitis B in China are reported mainly in adults, who have become the primary population of new HBV infections over 20 years of age [10]. In addition, in the USA, the population aged 25 to 44 are the highest proportion of new HBV infections [11]. In 2011, the guidelines for adult hepatitis B vaccination were issued by the Chinese Center for Disease Control and Prevention (CDC) and the Chinese Preventive Medicine Association (CPMA), recommending that all unvaccinated adults, especially those at high risk for HBV infection, should be targets for vaccination [12]. The National Demonstration Areas in China for Integrated Control of Infectious Diseases promoted hepatitis B vaccination for adults as one of the essential interventions as well. However, the need for booster immunization among adults is still controversial. Some studies have shown that, in adults, the level of anti-HBs produced by hepatitis B vaccination decreased over time and even lost its protective effect completely [13]. Several long-term studies of immune persistence have shown that the anti-HBs ≥ 10 mIU/mL proportion of adults immunized with hepatitis B vaccine after 20–30 years was 50–60%, but booster immunization was not recommended [14,15,16]. However, in China, according to a cohort study, the seroprotection rate of adult vaccination against hepatitis B was reduced to 58.3% after eight years [7].

Therefore, in order to improve China’s hepatitis B immunization strategy and the control of HBV infection in adults, it is crucial to consider long-term persistence after vaccination to determine whether a booster dose is needed. This study monitored the persistence of immunity after adults received full course of vaccination against hepatitis B in 1, 5 and 10 years to understand the immune effects of hepatitis B vaccination and support the implementation of immunization strategy against HBV in adults.

## 2. Materials and Methods

### 2.1. Study Setting and Design

Our study was carried out in Chaoyang District, Beijing. This district is an important commercial and cultural center with over 3.4 million residents, far exceeding some European capitals such as Paris. It is commercially developed, with the well-known Sanlitun district and several large shopping centers. In addition, Chaoyang District is highly internationalized, being home to 70% of Beijing’s foreign-related resources. As a key area of Beijing, it is also one of the National Demonstration Areas in China for the Integrated Control of Infectious Diseases. In this district, screening of hepatitis B in the general population has been conducted since 2010. Free vaccination against hepatitis B was offered for people who were negative for hepatitis B virus surface antigen (HBsAg), antibody against hepatitis B virus surface antigen(anti-HBs) and antibody against hepatitis B virus core antigen(anti-HBc) after screening. For the vaccination of these people, the type of vaccine was 20 μg Hepatitis B Vaccine Made by Recombined DNA Techniques in Yeast, and all vaccines were applied by provincial fixed channels in accordance with the regulation of products and stored by the designated community health service centers in accordance with the cold chain management requirements. To evaluate the immune persistence of the hepatitis B vaccine, our study conducted a cross-sectional serological survey of people aged ≥18 years after hepatitis B vaccination for 1, 5 and 10 years.

Three groups were set for 1 (Y1), 5 (Y5) and 10 (Y10) years after the full course of vaccination. The communities included in Chaoyang District were divided into urban and rural strata, and two communities in each stratum were selected to recruit subjects. Based on the published results of adult hepatitis B immune persistence studies, the sample size was estimated using the expected anti-HBs positive rates of 80%, 70% and 60% for Y1, Y5 and Y10, respectively [7,17,18]. Calculated from the sample size formula, at least 125, 164 and 188 subjects were included in Y1, Y5 and Y10 groups. Considering the accuracy and funding, 200 subjects were finally recruited in each group.

Through telephone interviews, subjects were recruited by trained investigators. Questionnaires and serological tests for hepatitis B were administered after subjects signed an informed consent form.

### 2.2. Participants

When recruiting study subjects, we first screened the immune system, and those aged ≥ 18 years and who been completely immunized with 20 μg recombinant hepatitis B vaccine in adulthood according to the standard procedure (0–1–6 months) were enrolled.

### 2.3. Data Collection

Questionnaires and blood samples were collected from the subjects. The questionnaires were administered by trained investigators. The following data were collected via questionnaires, including sociodemographic information of subjects and hepatitis B vaccination history. To avoid errors due to recall, immunization history was found in the records of immune system and completed by the investigators.

Fasting venous blood samples of 5 mL were collected from each subject and serum was separated. HBV markers (HBsAg, anti-HBs and anti-HBc), were tested by Abbott chemiluminescence detection method (I2000) and quantified by Chemiluminescence Microparticle Immuno Assay (CMIA).

As generally accepted, the positive determination criteria is HBsAg ≥ 0.05 IU/mL, anti-HBs ≥ 10.00 mIU/mL and anti-HBc ≥ 1.00 S/CO. Seroprotection was defined as an anti-HBs ≥ 10 mIU/mL. People who are positive for anti-HBs are considered immune protected.

### 2.4. Statistical Analysis

A statistical analysis was developed using the SPSS software program, version 21. We used descriptive statistics to calculate the means, geometric means, percentages and confidence intervals (CI). Comparison between the groups was used some appropriate tests (χ^2^ test, χ^2^ for trend, Fisher exact test and ANOVA test). In addition, Kruskal–Wallis test and Kolmogorov–Smirnov test were used to compare the GMC. The values of *p* < 0.05 were considered statistically significant.

The decrease of anti-HBs positive rates and GMC within the different groups were calculated by the mean annual decrease rate with the following formula: Mean annual decrease rate = 1 − $\sqrt[{n}]{B/A}$ (*n*: number of years elapsed; B: final data after *n* years; A: starting data).

## 3. Results

### 3.1. Demography and Hepatitis B Virus Serological Test Results of Participants

In all subjects, there were 253 (42.2%) males and 347 (57.8%) females, and the mean age of all individuals was 52.8(95%CI, (51.6,53.8)). According to gender, there was no significant difference between the subject groups (χ^2^ = 1.5, *p* = 0.475). The mean age (46.1, 49.7 and 46.5 years old, respectively) of the subjects in three groups was balanced when they completed the vaccination against hepatitis B.

In Table 1, the incidence of HBV infectious events over time is reported. Among all 600 subjects, the positive rates of HBsAg, anti-HBc and anti-HBs were 0, 2.5% (15/600) and 70.5% (423/600), respectively. Of the individuals who were positive for anti-HBc, 12 subjects were both positive for anti-HBs and anti-HBc and 3 subjects were single positive for anti-HBc.

### 3.2. Immune Persistence

**Figure 1:**
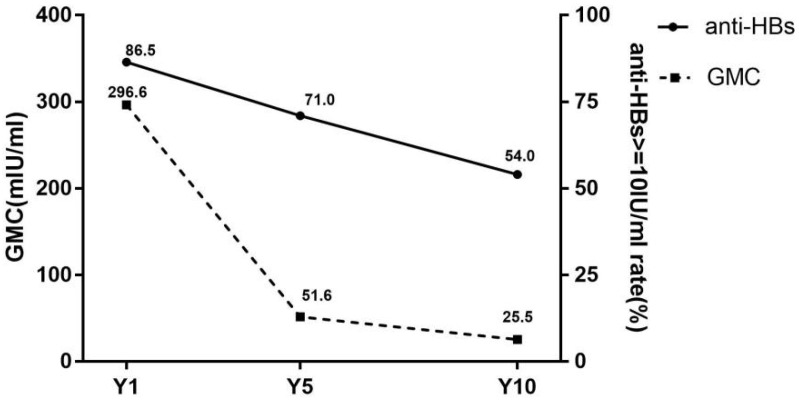
Seroprotection rates and GMC of subjects in different groups. anti-HBs, antibody against hepatitis B virus surface antigen; GMC, geometric mean concentration of anti-HBs; Y1, the group for 1 year after the full course of vaccination; Y5, the group for 5 years after the full course of vaccination; Y10, the group for 10 years after the full course of vaccination. Seroprotection rates representation of the anti-HBs positive rates (anti-HBs ≥ 10 mIU/mL).

The dynamic changes of seroprotection rates and GMC are shown in Figure 1. The seroprotection rates in the Y1, Y5 and Y10 groups were 86.5% (173/200), 71.0% (142/200) and 54.0% (108/200), respectively. The significant differences in seroprotection rates were revealed between three groups (χ^2^ = 50.8, *p* < 0.001). Furthermore, by χ^2^ for trend (trend χ^2^ = 50.7, *p* < 0.001), it was suggested that the seroprotection rate was decreased over time after vaccination.

The GMC was 296.6 mIU/mL, 51.6 mIU/mL and 25.5 mIU/mL at group Y1, Y5 and Y10, respectively, with statistically significant differences (H = 64.8, *p* < 0.001).

**Figure 2:**
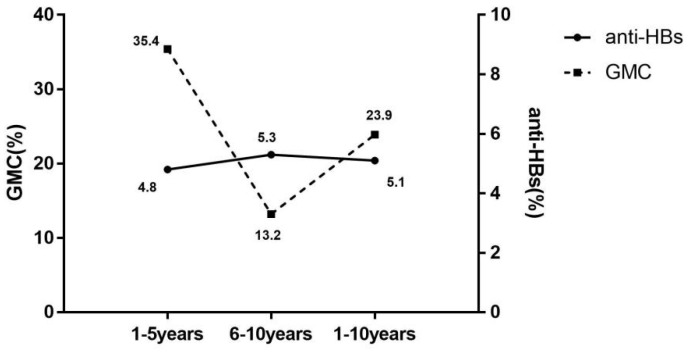
Mean annual decrease rate of seroprotection rate and GMC with the duration of immunization. anti-HBs(%), the mean annual decrease rate of antibody against hepatitis B virus surface antigen (mean annual decrease rate of seroprotection rate); GMC(%), the mean annual decrease rate of geometric mean concentration of anti-HBs; 1–5 years, 1–5 years after the full course of vaccination; 6–10 years, 6–10 years after the full course of vaccination; 1–10 years, 1–10 years after the full course of vaccination.

Further analysis of decay of anti-HBs levels with time of vaccination was performed according to serological results, in Figure 2. The mean annual decrease rate in seroprotection (5.1% per year) was significantly lower than the GMC (23.9% per year). The rate of decline in seroprotection remained at 5% per year for both 1–5 years and 5–10 years. Conversely, the mean annual decrease rate of GMC was significantly higher for 1–5 years than for 6–10 years, with the mean annual decrease rate in years 1–5 2.7 times higher than in years 6–10.

### 3.3. Effectiveness of Immunization against Hepatitis B of Adults in Different Age

**Figure 3:**
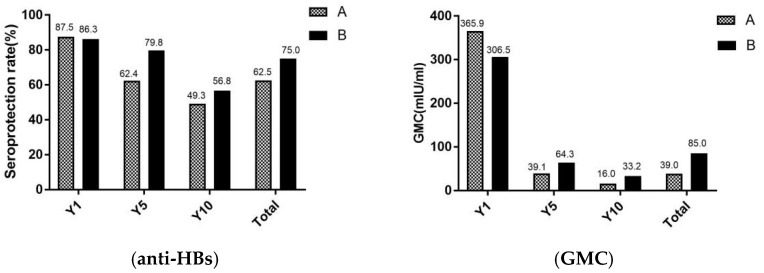
Seroprotection rate and GMC of adults in different age. A, the group A (adults who aged ≥ 60 years); B, the group B (adults who aged < 60 years); Y1, the group for 1 year after the full course of vaccination; Y5, the group for 5 years after the full course of vaccination; and Y10, the group for 10 years after the full course of vaccination. The seroprotection rate of representation of the anti-HBs positive rates (anti-HBs ≥ 10 mIU/mL), GMC representation of the geometric mean concentration of anti-HBs.

In Figure 3, the seroprotection rates and GMC of adults were revealed in different age. Total of study participants, the seroprotection rates and GMC were significantly lower in group A (aged ≥ 60 years) than in the group B (aged < 60 years) (χ^2^ = 10.4, *p* < 0.001) (Z = 1.6, *p* = 0.011). For seroprotection rates after vaccination against hepatitis B for 1 year, there were no significant differences between group A and group B. However, after the vaccination against hepatitis B for 5 and 10 years, the seroprotection rates were higher in group B than group A.

## 4. Discussion

There was evidence that simultaneous hepatitis B vaccination in neonates, adolescents and adults at once was the most effective strategy to control the HBV infection [19]. Thus, it is necessary to gradually implement hepatitis B vaccination for people other than newborns on the basis of current policies [12]. In order to provide a reference for further improvement immunization strategies for adults against hepatitis B, the immune persistence of adults for different years after hepatitis B vaccination were analyzed in our study.

In our study, no people with positive HBsAg were found, while the positive rate of anti-HBc was 2.5% with an increasing trend over time of immunization, which was similar to the previous study of 8 years after hepatitis B immunization with a 2.0% positive rate of anti-HBc [20]. In Shandong, one study showed that with five years after adult vaccination with the 20 μg recombinant hepatitis B vaccine, the positive rate of anti-HBc was 3.54%, and the other study showed that for nine years after immunization, this rate was 2.32% [18,21]. These positive rates of HBsAg and anti-HBc in the immunized population were significantly lower than the general population (2.4% and 15.7%), compared with antibody surveillance data of general adult in the same region at the same time. Therefore, adult immunization is highly effective in controlling infections, the free adult hepatitis B vaccination policy is worth promoting in China.

There have been some relevant studies of immune persistence of hepatitis B in adults. The seroprotection rate was over 60% after five years and declined to 58.3% after eight years, which similar to the present study [7,22]. Compared to other studies, the seroprotection rates in our results appear to be lower. In China, a large cohort study showed that the rate of adults aged 18–25 years with anti-HBs ≥ 10 mIU/mL was 98.3% after two years, with a full course of hepatitis B vaccine following a 0–1–6 schedule [23]. Another similar study conducted by W.W. pointed that the seroprotection rate of anti-HBs among adults was 92.3% after five years [24]. The reason for the difference between these studies and our study may be the age difference of the study subjects, which proved that vaccination is more effective in younger adults [25]. In addition, according to studies in Belgium and Alaska, the vaccination of adults against hepatitis B provided durable protection for up to 30 years, with seroprotection rates of 89.3% and 51.0% at 15 and 30 years, respectively [14,26]. Regarding immune memory after hepatitis B vaccination in adults, studies in Canada and other places have shown that it can be maintained for 20–30 years [14,15]. From these conclusions, we assumed that observed years of studies on the durability protection of adult hepatitis B vaccine in China were shorter than that of other developed countries. More research needs to be conducted in China on the need for booster immunization in adults as a way to explore hepatitis B immunization strategies.

Some studies, which had the same conclusion with this study, have shown that the immune effect of hepatitis B vaccine gradually decreased with increasing age [25]. In a recent study in China, seroprotection rates of 98.0% and 81.8% were observed in children who received the hepatitis B vaccine after 1 year and 10 years [27]. For another study in Italy, the seroprotection rate for neonates was 77.0%, 17 years after hepatitis B vaccination [28]. Thus, in the present study, the seroprotection rates in adults were significant lower than that in neonates or children and the mean annual decrease rate in seroprotection (around 5.0%) was higher than neonates (1.9%) [27]. In addition, compared to immunization effects according to different age groups in adults, the seroprotection rate in people over 60 years of age was already below 50.0% after 10 years of immunization, with a higher decline in GMC (95.6%) than in people under 60 years of age (89.2%).

In our results of anti-HBs positive rates and GMC, the anti-HBc positive cases have been excluded; the effect on GMC due to infection can therefore be excluded. In accordance with this study, with a time extension, adults were found to lose immune protection at a high proportion. The seroprotection rates of adults with hepatitis B vaccine declined rapidly after immunization, with 29.0% and 46.0% of subjects losing immune protection after 5 and 10 years. People with a single anti-HBc are considered to be previously infected with hepatitis B [1]. Although no people with positive HBsAg were found, people with anti-HBc alone were all in group Y10, which proved that the risk of hepatitis B in adults increased with a longer duration of vaccination. Meanwhile, in terms of GMC levels, it showed a decreasing trend, with the mean annual decrease rate in 1–5 years being 2.7 times faster than 6–10 years. GMC declined by more than 90.0% after 10 years of immunization, which substantially reduced the efficacy of vaccine protection and increased the risk of HBV breakthrough infection. A high proportion of HBV-susceptible individuals were adults, who were more likely to be exposed to risk factors and have a high risk of HBV infection [29,30]. In addition, with an advancing age, the ability of population to maintain immune memory decreased [31]. Therefore, it is recommended to focus on hepatitis B vaccination and vaccine immunization in adults. Early vaccination is recommended for adults over 30 years (not in immunization program) who have not been vaccinated. For adults at high risk for HBV, such as health care workers and homosexuals, screening 5–10 years after hepatitis B vaccination is recommended, as well as the development of relevant measures such as booster immunization for the unprotected population as early as possible.

## 5. Conclusions

In summary, adults who accepted a full course of vaccination against hepatitis B gained a high proportion of immune protection and reduced the risk of infection, but the seroprotection rate and level of GMC decreased quickly over time. Apart from this, the effectiveness and persistence of immunization in adults was lower than that in newborns and children, and adults who lose immune protection are still at risk of infection. Therefore, detection of HBV markers may be considered 5–10 years after full course of vaccination in adults, and booster immunization with hepatitis B vaccine is recommended for those found to be below protective levels.

## Figures and Tables

**Table 1:** Demography and HBV serological test results for all study subjects.

Group	*n*	HBsAg ≥ 0.05 IU/mL		Anti-HBs ≥ 10 IU/mL		Anti-HBc ≥ 1.00 S/CO	
		*n*	%	*n*	%	*n*	%
Y1	200	0	0	173	86.5	2	1.0
Gender							
Male	78	0	0	63	80.8	1	1.3
female	122	0	0	110	90.2	1	0.8
age (95%CI)	47.1 (45.3, 49.0)	-		46.4 (43.5, 48.9)		36.5 (36.0, 37.0)	
Y5	200	0	0	142	71.0	6	3.0
Gender							
Male	85	0	0	59	69.4	5	5.9
female	115	0	0	83	72.2	1	0.9
age (95%CI)	54.7 (52.6, 56.8)	-		54.0 (51.2, 56.8)		64.5 (61.0, 68.0)	
Y10	200	0	0	108	54.0	7	3.5
Gender							
Male	90	0	0	43	47.8	5	5.6
female	110	0	0	65	59.1	2	1.8
age (95%CI)	56.5 (55.1, 57.8)	-		55.8 (54.0, 57.7)		54.5 (53.0, 56.0)	
Total	600	0	0	423	70.5	15	2.5
Gender							
Male	253	0	0	165	65.2	11	6.7
female	347	0	0	258	72.3	4	1.6
age (95%CI)	52.8 (51.6, 53.8)	-		50.9 (49.7, 52.3)		57.7 (50.8, 64.3)	

## Data Availability

The data presented in this study are available upon request from the corresponding author. The data are not publicly available due to privacy.
